# Nutritional Strategies and Aging: Current Evidence and Future Directions

**DOI:** 10.3390/molecules31050756

**Published:** 2026-02-24

**Authors:** Serena Castelli, Gilda Aiello, Vincenzo Aiello, Elena Massimino, Mattia Pieri, Isaac Amoah, Mauro Lombardo, Gianluca Tripodi, Sara Baldelli

**Affiliations:** 1 Department for the Promotion of Human Science and Quality of Life, San Raffaele Open University, Rome, Via di Val Cannuta, 247, 00166 Rome, Italy; serena.castelli@uniroma5.it (S.C.); gilda.aiello@uniroma5.it (G.A.); vincenzo.aiello@uniroma5.it (V.A.); elena.massimino@uniroma5.it (E.M.); mauro.lombardo@uniroma5.it (M.L.); gianluca.tripodi@uniroma5.it (G.T.); 2 IRCCS San Raffaele Roma, 00166 Rome, Italy; 3 Independent Researcher, Milano Marittima, 48015 Emilia Romagna, Italy; nutrizionistamattiapieri@gmail.com; 4 Department of Biochemistry and Biotechnology, Kwame Nkrumah University of Science and Technology, Kumasi 00233, Ghana; isaacamoah@knust.edu.gh

**Keywords:** aging, oxidative stress, nutrition-based interventions, nutraceuticals, mitochondrial health, disease prevention

## Abstract

Aging is a progressive degenerative process characterized by the depletion of tissue stem cell reserves, organ atrophy, sarcopenia, and an impaired capacity to respond to physiological stress and injury. These changes lead to a reduction in both overall life expectancy and disease-free lifespan. Since aging represents a major risk factor for numerous diseases, including neurodegenerative, cardiovascular, and metabolic disorders, recent research has increasingly focused on identifying effective intervention strategies to promote “healthy aging” by slowing down the aging process as much as possible. At the molecular level, multiple factors contribute to cellular aging and, consequently, to the onset of senescence. These include mitochondrial dysfunction, defective DNA repair mechanisms, epigenetic reprogramming, and chronic low-grade inflammation. Among the mechanisms driving cellular senescence, oxidative stress is recognized as a key contributor to the loss of replicative capacity. When reactive oxygen species (ROS) levels exceed a critical threshold, they can damage essential macromolecules, including DNA. Therefore, ROS and oxidative stress represent crucial therapeutic targets to be considered in strategies aimed at counteracting cellular senescence. Based on these causal factors, several strategies have been identified that target modifiable lifestyle determinants, with a primary focus on nutrition and nutraceutical interventions. In this context, the present review aims to critically analyze scientific evidence regarding nutritional approaches designed to slow down the aging process, including their effects at the molecular level. Specifically, these strategies aim to reduce inflammation, preserve mitochondrial function to modulate ROS production, and protect macromolecules from oxidative stress.

## 1. Introduction

Although chronological age is, at least currently, unchangeable, numerous strategies can modulate biological age, thereby influencing the aging process and promoting healthy aging. These approaches primarily target the hallmarks of aging and emphasize lifestyle-based preventive interventions, with nutrition and exercise being the most extensively studied. Because aging is a gradual, long-term process that becomes clinically evident only at advanced stages—when many mechanisms are already established—both clinical practice and translational research largely rely on preventive strategies. Indeed, many aging-related mechanisms are irreversible, making the prevention of their onset the most effective therapeutic approach.

Aging is a physiological process characterized by a progressive and irreversible decline in systemic function, affecting both somatic and cognitive domains. This phenotype arises from the cumulative accumulation of molecular alterations, including subtle but persistent functional perturbations and the aberrant consequences of minor molecular errors. A paradigmatic example of this molecular drift is physiological telomere shortening, which significantly contributes to age-associated cellular dysfunction. At the molecular level, these processes converge on cellular senescence, primarily characterized by reduced proliferative capacity and stable cell cycle arrest [1].

Senescent cells exhibit distinct phenotypic markers, including upregulation of senescence-associated β-galactosidase (SA-β-Gal), secretion of the senescence-associated secretory phenotype (SASP), formation of senescence-associated satellite distensions (SADS), increased expression of cyclin-dependent kinase inhibitors (CDKIs) such as p53, p21, and p16, and reduced lamin B1 expression [2]. Although cellular senescence is among the most intensively studied hallmarks, aging is now recognized as a multifactorial process involving additional hallmarks spanning molecular, cellular, and systemic levels.

Many of these hallmarks are tightly linked to cellular metabolism and its biochemical regulation, including mitochondrial dysfunction, impaired proteostasis, and epigenetic dysregulation, which integrates metabolic status with gene expression control. Aging is further characterized by genomic instability and telomere attrition, increasing susceptibility to DNA damage. These alterations not only promote cellular senescence but also lead to a progressive decline in stem cell function, compromising tissue and organ homeostasis. At the systemic level, aging is associated with altered intercellular communication, chronic low-grade inflammation (*inflammaging*), and dysregulation of nutrient-sensing pathways [3].

The functional consequences of aging, including sarcopenia—defined as the progressive loss of muscle mass and strength—represent major contributors to frailty. Aging-related hallmarks predispose individuals to a wide range of pathological conditions, including musculoskeletal disorders, increased fracture risk, malignancies linked to genomic instability, gastrointestinal diseases, and neurodegenerative disorders such as Alzheimer’s disease [4,5]. Consistent epidemiological evidence has led to the classification of many of these conditions as age-related diseases, highlighting the central role of aging in increasing disease susceptibility [4].

In this framework, a distinction is made between chronological aging and biological aging, the latter reflecting the functional state of the organism. These two dimensions often diverge, with biological age exceeding chronological age in many individuals, indicating accelerated aging [3,6,7]. This discrepancy has fueled growing scientific interest in strategies capable of modulating biological aging. Accumulating evidence indicates that nutritional strategies and nutraceutical interventions can beneficially influence multiple hallmarks of aging, including chronic inflammation, oxidative stress, mitochondrial dysfunction, and nutrient-sensing pathways. Owing to their systemic, cellular, and molecular effects, these approaches represent sustainable, long-term tools to promote healthy aging and reduce the risk of age-related diseases. Accordingly, this review summarizes the nutritional strategies currently identified as effective in the management of aging.

## 2. Biochemical and Biological Basis of Aging

Aging is a physiological process characterized by the progressive functional decline of the organism. During this process, an individual’s susceptibility to age-related diseases gradually increases; therefore, although aging itself is not a disease, many conditions are considered aging-associated. Aging, in fact, promotes a state of frailty, which is defined based on how an individual’s characteristics match established frailty criteria [8]. Many of these criteria correspond to typical aging-related events and indicate not only the individual’s propensity to develop diseases but also the relative risk regarding their response to therapies [9].

For this reason, aging itself represents a topic of particular scientific interest, as it carries significant biological, social, and economic implications. Indeed, the increase in average lifespan exerts considerable pressure on healthcare systems, especially when not accompanied by healthy aging. In order to understand the biological and biochemical foundations of aging and to enable proper management and prevention, the field of biogerontology has emerged. Biogerontology focuses on studying the molecular and cellular mechanisms underlying aging with the aim of promoting healthy longevity. 

At the molecular level, a key feature of aging lies in the accumulation of reactive oxygen/nitrogen species (ROS/RNS), which can act as damaging molecules by attacking macromolecules, including DNA itself.

**Figure 1:**
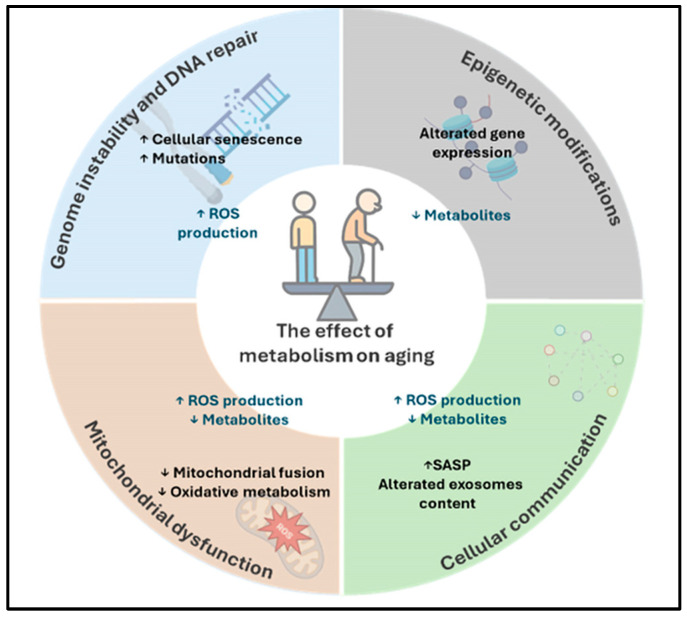
At the molecular level, aging is characterized by the progressive accumulation of ROS, which act as damaging agents by targeting cellular macromolecules, including DNA. Classifying the hallmarks of aging into three main categories—primary, antagonistic, and integrative—twelve distinct hallmarks can be identified, many of which are closely associated with elevated levels of ROS and oxidative stress. Additionally, senescent cells produce proinflammatory and matrix-degrading molecules in what is known as the senescence-associated secretory phenotype (SASP).

When grouping the hallmarks of aging into three categories, namely primary, antagonistic, and integrative, 12 hallmarks can be defined, many of which are linked to increased ROS levels and oxidative stress (Figure 1) [10].

Both ROS and RNS act as important signaling molecules capable of regulating gene expression through their interaction with transcription factors that are sensitive to these reactive species [11]. Many of these transcription factors are responsible for activating protective cellular responses. For example, cells may trigger hypoxic or pseudo-hypoxic pathways (through the activation of HIF-1α) to reduce mitochondrial metabolism and thereby limit further ROS production, or they may induce cell-cycle arrest or apoptosis (by activating p53) when the levels of these highly reactive molecules become too high to be managed.

In the context of aging, the accumulation of ROS/RNS occurs gradually, and over time these molecules typically cause damage to DNA (genome instability), to organelles (increased autophagy), or to cellular membranes (lipotoxicity). The site of ROS/RNS production within cells is crucial for determining their biological effects. These species can be generated endogenously by mitochondria, the endoplasmic reticulum (ER), and peroxisomes, or they can arise exogenously in response to exposure to external factors such as pollutants, radiation, food components, medications, or drugs [12]. Among the main targets of oxidative damage is DNA, which can consequently undergo replication errors, chromosome segregation defects, oxidative modifications, and spontaneous hydrolytic reactions, ultimately leading to genome instability. Genome instability, in addition to being driven by increased ROS/RNS levels, can also arise from both endogenous and exogenous sources. The resulting damage includes point mutations, deletions, translocations, telomere shortening, single- and double-strand breaks, chromosomal rearrangements [10]. In addition to the increase in DNA damage, aging is also characterized by a reduced capacity for DNA repair, which leads to the progressive accumulation of these mutations.

Alongside this, aging is accompanied by a chronic, low-grade inflammatory state known as inflammaging, which is a central biological hallmark of aging and a key driver of age-related functional decline. Inflammaging results from the progressive accumulation of senescent cells, which secrete pro-inflammatory mediators as part of the SASP, as well as immunosenescence, mitochondrial dysfunction, and increased activation of innate immune pathways [13]. At the molecular level, persistent stimulation of inflammatory signaling cascades, including the NF-κB and NLRP3 inflammasome pathways, leads to sustained production of cytokines such as IL-6, TNF-α, and IL-1β, contributing to tissue damage, impaired regeneration, and metabolic dysregulation [14]. In parallel, age-related alterations in intestinal barrier integrity and microbiota composition further amplify systemic inflammation through increased translocation of microbial-derived inflammatory signals [15,16]. Overall, this chronic inflammatory environment not only accelerates biological aging but also plays a causal role in the pathogenesis of sarcopenia, insulin resistance, cardiovascular disease, and neurodegeneration, positioning inflammation as a central therapeutic target in strategies to promote healthy aging [17,18].

### 2.1. Genome Instability and DNA Repair in Aging

Genomic instability is the physiological tendency of the genome to accumulate errors and mutations over time. The increase in such mutations is driven by two key events: the accumulation of mutations caused by exogenous factors (elevated ROS levels, exposure to mutagenic agents, lifestyle factors) and the decline in the efficiency of DNA repair mechanisms (Figure 1) [19]. External events, including endogenous genotoxins such as ROS or aldehydes, together with exogenous genotoxic agents such as environmental chemicals, UV radiation, or X-rays, can induce various types of DNA damage (such as different classes of strand breaks, nicks, gaps, abasic sites, adducts, crosslinks, and other chemical modifications). These lesions can lead to three possible outcomes: successful damage resolution, apoptosis, if the damage is extensive and irreparable, or cellular senescence—a state in which the cell permanently loses its proliferative capacity without undergoing cell death. Cellular senescence represents the primary molecular driver of aging [20].

Both nuclear and mitochondrial DNA undergo similar processes of mutation accumulation. With respect to mitochondrial DNA, the primary mutagenic drivers are ROS, as mitochondria represent the major source of cellular ROS. Among the various mutagenic stimuli, ROS constitute a key link between the molecular alterations associated with aging and the biochemical–metabolic changes that accompany it. Notably, one of the hallmarks of senescent cells is the loss of mitochondrial functionality. Mitochondria become less efficient, generating reduced amounts of energy while releasing increased levels of ROS. In addition, senescent cells exhibit suppressed antioxidant defenses, which are insufficient to buffer excessive ROS levels, thereby enabling the development of oxidative stress [21]. The consequences of DNA damage can vary widely. The insertion of a mutation does not always lead to a detectable protein-level effect; some mutations are phenotypically neutral. Others, however, alter the function of the protein encoded by the affected gene and can therefore produce downstream effects, including reduced functional efficiency or proteostatic stress, particularly when protein structure or activity is compromised [20,22]. In many cases, genomic instability has been identified as a primary driver of aging precisely because DNA alterations lead to functional impairments at the protein level, as well as reduced proteome stability. These effects contribute to numerous age-associated pathologies, including diseases caused by protein misfolding and aggregation, such as Parkinson’s and Alzheimer’s disease [23]. Ultimately, as with any complex multivariate phenomenon, it is extremely challenging to identify a single key driver, since it is difficult to determine which process fundamentally precedes and influences the others. From this perspective, oxidative stress can also be considered a central component of the aging process, as it directly contributes to DNA damage and represents a major determinant of numerous aging-related pathologies, including neurodegenerative diseases [23].

A further molecular consequence of the age-associated accumulation of mutations is telomere shortening. In mammals, telomeres consist of long arrays of TTAGGG repeats bound by shelterin, a specialized protein complex that protects chromosome ends from being recognized as DNA breaks. Due to the end-replication problem and incomplete lagging-strand synthesis, telomeres progressively erode with each cell cycle. In germline cells and in specific somatic stem cell compartments, telomerase counteracts this erosion by adding back telomeric repeats. However, telomerase expression is developmentally silenced in the vast majority of somatic tissues, thereby imposing a finite proliferative potential [22].

As telomeres shorten beyond a critical threshold, shelterin becomes insufficient to mask chromosome ends, which are subsequently detected as persistent DNA double-strand breaks. This triggers a chronic DNA damage response (DDR), primarily involving ATM/ATR signaling, ultimately enforcing a stable state of replicative senescence. Importantly, telomere dysfunction not only limits regenerative capacity, but also contributes to a pro-inflammatory SASP, thereby amplifying tissue-level aging and promoting multiple age-related disorders, including fibrotic diseases, metabolic dysfunction, and neurodegeneration [24].

Although telomere attrition was long considered an unmodifiable aspect of aging, recent research has revealed that lifestyle factors significantly influence the rate of telomere shortening, indicating that this process is both preventable and modifiable [25]. Indeed, lifestyle interventions—particularly adherence to a Mediterranean-style diet and regular physical activity—have been shown not only to slow telomere erosion but also to increase telomere length, regardless of the initial baseline length [26].

### 2.2. Epigenetic Alterations in Aging

An essential component of the aging process, and one that offers significant opportunities for intervention, is epigenetics. Epigenetic mechanisms encompass non-heritable modifications that alter the transcriptional capacity of the genome by promoting or silencing gene expression without changing the underlying DNA sequence. Epigenetic alterations are highly prevalent during aging and are strongly influenced by lifestyle factors [19]. Epigenetic variations are strongly influenced by the bioavailability of the substrates required for these modifications. Such substrates originate from metabolic reactions and are therefore tightly linked to the cell’s bioenergetic state and to the quality and quantity of energy sources available to the cell. 

Epigenetic alterations can occur at multiple levels, including histone modifications, non-histone protein modifications, and changes involving non-coding RNAs. Histone modifications consist of post-translational changes to histone proteins that can alter their charge and conformation, thereby influencing their interaction with DNA and modulating chromatin compaction. These structural changes ultimately affect the accessibility of the transcriptional machinery and, consequently, gene expression patterns (Figure 1).

Several types of histone modifications have been described, including methylation, acetylation, phosphorylation, ubiquitination, and ADP-ribosylation, each exerting distinct effects on gene expression. However, a unifying or universal pattern of histone modification changes during aging has not yet been established [27]. In general, these alterations appear to be highly context-dependent, varying according to the specific chromatin region involved. Moreover, histone modifications during aging often occur in a stochastic manner, leading to heterogeneous and individualized transcriptional outcomes.

Histone methylation can exert either activating or repressive effects on transcription, depending on the specific residue and methylation state involved. H3K4me3, a well-established marker of active gene transcription, plays a particularly relevant role in aging, as it has been detected on the promoters of genes associated with senescence and age-related pathways. This modification has been consistently linked to aging across multiple experimental systems, including *C. elegans* [28,29,30], hematopoietic stem cells (HSCs) [29], and neurons. 

Global alterations in H3K27me3 have also been shown to influence lifespan across several organisms. H3K27me3 is typically associated with transcriptional repression and the maintenance of compacted heterochromatin, primarily through the activity of Polycomb Repressive Complex 2 (PRC2), which catalyzes the trimethylation of lysine 27 on histone H3. This modification plays a central role in preserving cellular identity by stabilizing long-term gene silencing programs, particularly those that regulate developmental pathways [31].

Altered H3K27me3 landscapes have been observed in aging stem cell populations, such as hematopoietic stem cells and neural stem cells, where disrupted PRC2 activity impairs self-renewal, biases differentiation, and reduces tissue homeostasis. In model organisms, including *Drosophila* [32] and *C. elegans* [33], genetic perturbation of PRC2 components or enzymes regulating H3K27 methylation significantly impacts lifespan, underscoring the importance of this epigenetic mark in maintaining organismal longevity.

Histone methylation is a reversible post-translational modification in which histone methyltransferases (HMTs) add one to three methyl groups to lysine or arginine residues using S-adenosyl-L-methionine (SAM) as the methyl donor. The reaction produces S-adenosyl-L-homocysteine (SAH), a potent feedback inhibitor, thereby tightly coupling chromatin methylation to cellular methyl-metabolism [34].

Compared with histone methylation, histone acetylation shows a clearer and more consistent association with aging. Acetylation is mediated by lysine acetyltransferases—particularly histone acetyltransferases (HATs)—which add acetyl groups to lysine residues on histone tails, whereas histone deacetylases (HDACs) remove them. The activity of these enzymes is tightly coupled to cellular metabolic status because acetylation reactions depend on the availability of acetyl groups, primarily provided by mitochondrial acetyl-CoA. Thus, when energetic and metabolic fluxes are high, intracellular acetyl-CoA levels increase, enhancing the pool of substrates for histone acetylation and other chromatin-related modifications. This direct biochemical link between metabolism and chromatin regulation underscores how metabolic state shapes gene expression programs, a mechanism that plays a critical role in the aging process [35].

Among histone deacetylases, sirtuins—classified as class III HDACs—play a central role in aging due to their dependence on intracellular NAD^+^ levels and their broad impact on genome stability. Sirtuins catalyze the deacetylation of lysine residues in a metabolic state–dependent manner, thereby linking nutrient availability, mitochondrial function, and chromatin regulation.

Several studies have shown that SIRT1 expression declines with age in multiple tissues, including the liver, heart, kidney, brain, and lung, contributing to impaired stress responses and reduced genomic maintenance during aging [36,37]. SIRT6, in contrast, has emerged as a key regulator of telomere integrity: it promotes telomere stabilization through its deacetylase activity, and its overexpression has been shown to delay or even block the onset of cellular senescence, highlighting its potential role as a longevity factor [38].

Beyond histone-based epigenetic regulation, DNA epigenetic modifications also exert a major influence on the aging trajectory and its pathological outcomes. Among these, DNA methylation, primarily occurring at CpG islands, is one of the most widely studied hallmarks of epigenetic aging. Age-associated alterations in CpG methylation patterns have been consistently linked to increased vulnerability to age-related diseases, including cancer, neurodegeneration, and metabolic dysfunctions [39].

This tight association between methylation dynamics and biological aging has led to the development of epigenetic methylation clocks, quantitative models that capture the cumulative effect of DNA methylation changes across the genome. These clocks serve as robust biomarkers that reflect individual variability in biological aging rates, often outperforming chronological age in predicting disease risk and mortality [40].

In this context, dietary patterns can play a fundamental role, as they have been shown to modulate the cellular epigenome. Molina-Serrano et al. conducted an extensive review summarizing how different nutritional interventions influence histone PTMs, highlighting the sensitivity of histone acetylation and methylation states to nutrient availability and metabolic fluxes [41].

Similarly, Noro et al. examined the interplay between nutrition and DNA methylation patterns, demonstrating a strong association between daily intake of zinc and vitamin B3 and the levels of 5-methylcytosine (5mC) and 5-hydroxymethylcytosine (5hmC) across the genome [42]. These findings further support the notion that dietary micronutrients act as key metabolic cofactors in one-carbon metabolism and redox reactions, thereby shaping the epigenetic landscape and influencing the rate of biological aging.

### 2.3. Cellular Communication Mechanisms in Aging

In the context of the relationship between cellular biochemistry and the aging process, both inter-organelle and cell–cell communication play a central role, as they determine how intracellular events are translated into the systemic phenotypes that characterize organismal aging. Importantly, the biochemical state of the cell profoundly influences intercellular communication, since signaling relies on the release or exchange of lipids, metabolites, proteins, and nucleic acids—including ncRNAs—whose composition and abundance directly reflect the cell’s internal metabolic and regulatory status [43].

Thus, age-associated alterations in mitochondrial activity, redox balance, metabolic fluxes, and proteostasis are propagated beyond the individual cell through these messengers, ultimately shaping tissue homeostasis, inflammatory tone, and the progression of age-related dysfunction (Figure 1).

The secretome of senescent cells markedly differs from that of healthy, young cells. Although senescent cells are irreversibly arrested in the cell cycle, they remain metabolically active and secrete a wide array of signaling molecules that collectively constitute the SASP. This secretory activity propagates and amplifies the senescent phenotype within the surrounding tissue, influencing inflammation, extracellular matrix remodeling, and stem cell function [44].

The other side of the coin refers to metabolites with potential protective functions, which are produced in conditions of aging or cellular damage, as occurs with N-acetylaspartate, which is passively released following neuronal injury. In peripheral tissues, it is capable of enhancing resistance to atrophic stimuli, which are characteristic of the aging process [45].

A substantial portion of this communication is mediated by extracellular vesicles, including microvesicles and exosomes, which transport signaling lipids, proteins, metabolites, and regulatory RNAs. Through these vesicles, senescent cells can disseminate stress signals and altered molecular cargo to neighboring and distant cells, thereby contributing to tissue dysfunction and systemic aging.

It has been demonstrated that exosomal cargo changes dynamically during aging, and senescent cells release extracellular vesicles enriched in molecules capable of propagating senescence signals. In particular, senescence-associated exosomes contain specific microRNAs that promote the expression of pro-senescence genes in otherwise young or healthy cells, thereby accelerating the spread of cellular aging within tissues.

In addition, these exosomes transport pro-inflammatory cytokines, including IFN-α, TNF-α, IL-17, and IL-10, which contribute to chronic low-grade inflammation—one of the defining features of aging (inflammaging). Through this altered exosomal communication, senescent cells exert systemic effects that reinforce tissue dysfunction and age-related pathology [46].

The characterization of this relationship has not only enabled the identification of exosomes as biomarkers of aging, but has also raised the possibility that these vesicles could be harnessed to modulate and improve the aging process itself. By selectively manipulating exosomal cargo or engineering vesicles with defined molecular profiles, it may be possible to counteract pro-senescence signaling, attenuate chronic inflammation, or restore more youthful intercellular communication networks, as demonstrated in mesenchymal stem cells MSCs cells [47]. This emerging perspective positions exosomes both as diagnostic tools and as potential therapeutic agents in the context of healthy aging.

### 2.4. Mitochondrial Dysfunction

The triggers of the aging process, including genomic instability and oxidative stress, converge on a common target: the mitochondrion. As the central hub of cellular energy production, mitochondrial dysfunction leads to disrupted cellular metabolism. It is not possible to clearly distinguish cause from effect between the increased ROS production that characterizes aging—also responsible for the accumulation of mutations—and mitochondrial dysfunction, which itself leads to elevated intracellular ROS levels (Figure 1). This establishes a self-perpetuating vicious cycle in which ROS-induced damage impairs mitochondrial function, and increasingly dysfunctional mitochondria further enhance ROS generation, thereby exacerbating membrane damage, inflammation, and, in some cases, apoptosis [48,49]. Mitochondrial deterioration emerges as a key driver underlying many age-related pathologies, including cancer and neurodegenerative disorders [50].

The events that induce mutations in genomic DNA can also affect mitochondrial DNA, which in humans consists of a small double-stranded circular molecule of 16,569 bp encoding 37 genes, including 13 polypeptides, 2 rRNAs, and 22 tRNAs [51].

The resulting mitochondrial dysfunction leads to their recognition as targets for elimination by the mitophagic machinery. This process mitigates the detrimental effects that damaged mitochondria would exert on the cell if they were retained. Autophagic removal of dysfunctional mitochondria can occur through ROS-mediated mechanisms: ROS can directly activate mitophagy-related proteins (e.g., BNIP3 and Parkin) or indirectly stimulate specific MAPKs, consequently promoting mitophagic activation. Ultimately, mitophagy counterbalances mitochondrial dysfunction and slows cellular metabolic degeneration [52].

Whereas mitophagy, in this context, functions as an anti-aging process, alterations in mitochondrial dynamics (fusion/fission) can instead promote aging. Indeed, numerous lifestyle and dietary factors have been shown to directly influence the balance between mitochondrial fusion and fission. Mitochondrial fusion is strongly associated with proper organelle function and is a marker of increased mitochondrial membrane potential. Some examples of this relationship include intermittent fasting, which activates AMPK and inhibits mTOR, thereby promoting mitochondrial fusion and reducing fission [53]; similarly, high-intensity intermittent exercise exerts comparable effects by enhancing PGC-1α and AMPK activity [54]. Jia and colleagues summarized the involvement of altered mitochondrial dynamics in age-associated diseases, highlighting their association also with neurodegenerative diseases [49]. This close relationship has been therapeutically exploited, as several treatments used in aging-associated diseases—including Parkinson’s disease, Alzheimer’s disease, amyotrophic lateral sclerosis, and coronary disorders—specifically target mitochondria, aiming to enhance mitochondrial activity while reducing fission and organelle degeneration [49,51].

The functional consequences of mitochondrial alterations associated with aging lead to metabolic changes in senescent cells, resulting not only in impaired ATP production but also in variations in metabolite availability. Mitochondrially derived metabolites serve as key signaling molecules in both intra- and intercellular communication, and they are moreover responsible for post-translational modifications of proteins and histones, as well as of DNA itself [55]. Thus, the decline in mitochondrial function not only slows mitochondrial metabolic activity but also reduces the availability of key metabolites, including acetyl-CoA and α-ketoglutarate, which—when supplemented exogenously—may help ameliorate aging-related cellular conditions. For these reasons, targeting metabolic pathways has been proposed as a therapeutic approach not only for the treatment of established aging-related diseases but also for preventing aging and extending healthy lifespan [49,56].

## 3. Central Role of Oxidative Stress and ROS During Aging

**Figure 2:**
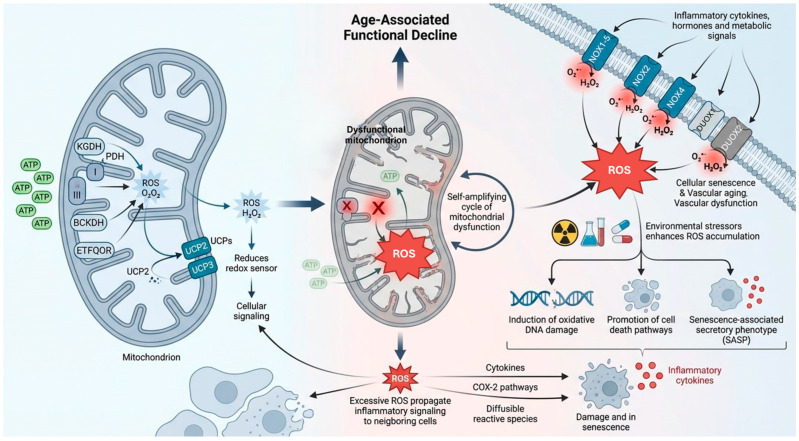
Mitochondrial dysfunction leads to increased ROS production and reduced ATP generation, establishing a self-amplifying cycle of oxidative damage. In parallel, NADPH oxidases activated by inflammatory and metabolic signals further contribute to ROS accumulation. Excessive ROS promote DNA damage, cellular senescence, inflammatory signaling and age-associated functional decline.

Aging is a multifactorial process resulting from the interaction between physiological decline and pathological factors, including chronic diseases. Among the central mechanisms involved, mitochondrial dysfunction plays a pivotal role, closely linking oxidative stress and inflammation, which act both as consequences and drivers of cellular aging. In this context, mitochondria function as regulatory hubs that integrate redox and inflammatory signals, coordinating age-associated cellular decline. Mitochondria and NADPH oxidases (NOX) represent the major intracellular sources of ROS [36]. While mitochondria generate ATP through oxidative phosphorylation, they are also a primary source of ROS, mainly produced at complexes I and III of the electron transport chain [57]. Additional mitochondrial enzymes, including α-ketoglutarate dehydrogenase (KGDH), pyruvate dehydrogenase (PDH), branched-chain ketoacid dehydrogenase (BCKDH), and ETFQOR, can significantly contribute to mitochondrial ROS (mtROS) generation (Figure 2) [57,58]. Under conditions of mitochondrial damage, ATP production declines while ROS levels increase, establishing a self-amplifying cycle of dysfunction [59]. Uncoupling proteins (UCPs), particularly UCP2 and UCP3, mitigate oxidative damage by reducing mitochondrial membrane potential and limiting excessive ROS formation [60,61,62]. UCP2 also acts as a redox sensor, participating in feedback regulation of mtROS production [63]. Conversely, pro-inflammatory cytokines such as TNF-α exacerbate mitochondrial dysfunction by impairing complex I activity, thereby promoting ROS accumulation, cell death pathways, and the SASP [64,65,66,67]. NOX enzymes are major contributors to ROS production in aging and age-related diseases, particularly within the cardiovascular system [68,69,70]. The NOX family includes seven isoforms (NOX1–5, Duox1, Duox2), which generate either superoxide (O_2_•^−^) or hydrogen peroxide (H_2_O_2_) in response to inflammatory cytokines, hormones, and metabolic signals [71,72,73]. Among them, NOX1, NOX2, and NOX4 are strongly implicated in cellular senescence and vascular aging, with NOX4 identified as a key regulator of inflammatory gene expression and vascular dysfunction during aging (Table 1) [74,75].

ROS accumulation is further enhanced by environmental stressors such as radiation, chemicals, and drugs, as well as by inflammatory and apoptotic processes [78]. Excessive ROS induce oxidative DNA damage and can propagate inflammatory signaling to neighboring cells through bystander effects, mediated by cytokines, COX-2 pathways, and diffusible reactive species [79,80]. Although controlled ROS production is essential for cellular signaling, excessive oxidative stress promotes mitochondrial damage and is considered a central mechanism of age-associated functional decline [81,82].

This concept underlies the mitochondrial theory of aging, which proposes that mtROS-driven mtDNA damage contributes to aging progression [83]. While recent studies in long-lived model organisms suggest a more complex relationship between mtROS and lifespan [84]. mtROS remain clearly implicated in the development of age-related pathologies [85]. Aging is also associated with redox imbalance and progressive weakening of antioxidant defenses, as supported by studies showing lifespan extension in mice with enhanced antioxidant capacity and reduced longevity in models lacking Copper-Zinc Superoxide Dismutase (CuZnSOD, SOD1) [86,87,88,89]. Nonetheless, conflicting evidence from invertebrate models indicates that oxidative stress alone may not fully account for aging [90]. Mitochondrial dynamics are profoundly altered during aging and are regulated by oxidative stress, intracellular calcium, and energy-sensing pathways. Increased intracellular Ca^2+^ levels activate Ca^2+^/calmodulin-dependent protein kinase, promoting Drp1 phosphorylation and mitochondrial remodeling [91,92,93,94]. Aging is also associated with reduced AMP-activated protein kinase (AMPK) activity, further disrupting the balance between mitochondrial fission and fusion and exacerbating oxidative stress and inflammation [95,96]. Environmental stressors such as UV radiation accelerate these alterations, contributing to tissue-specific aging processes [97]. The accumulation of dysfunctional mitochondria overwhelms mitophagy, reinforcing a vicious cycle of ROS production and cellular damage. Mitochondria also act as sources of pro-inflammatory signals through the release of ROS and mitochondrial DNA (mtDNA), which activate inflammasomes and cytosolic DNA-sensing pathways [98]. Age-related mitochondrial dysfunction is further amplified by endoplasmic reticulum (ER) stress, which impairs oxidative phosphorylation (OXPHOS), reduces ATP production, and increases ROS levels, particularly in aging cardiac tissue [76,77]. Pharmacological attenuation of ER stress, for example with 4-phenylbutyrate (4-PBA), has been shown to partially restore mitochondrial function in aged hearts [76]. Chronic low-grade inflammation represents another hallmark of aging [99,100]. Elevated levels of pro-inflammatory cytokines such as IL-1α, TNF-α, and IL-6 have been consistently observed in aged animal models and human tissues [101,102]. Aging-associated inflammation is further sustained by defective mitophagy and increased mtDNA release, which activate the cGAS-STING pathway [103,104,105]. Pharmacological enhancement of mitophagy, for instance with urolithin A, significantly reduces cGAS-STING activation and mitigates age-related inflammatory responses [105]. Inflammasome activation, particularly through the NLRP3 complex, represents a critical link between mitochondrial dysfunction and cellular senescence [106]. Inhibition of inflammasome signaling in NLRP3-deficient mice alters aging-related metabolic pathways and is associated with telomere preservation, suggesting a protective role against senescence [107]. Additionally, mitochondrial dysfunction in immune cells contributes to systemic aging: impairment of mitochondrial transcription factor A (TFAM) in T lymphocytes induces mitochondrial dysfunction and promotes chronic inflammation, accelerating aging phenotypes [108].

Collectively, these findings indicate that mitochondrial dysfunction, redox imbalance, and inflammation are tightly interconnected processes that converge to drive aging and age-associated diseases.

## 4. Therapeutic Targets Against Oxidative Stress

ROS and RNS play a central role in aging by promoting molecular damage, cellular senescence, and chronic inflammation. To counteract oxidative stress and limit age-related degeneration, cells rely on integrated enzymatic and non-enzymatic antioxidant systems that maintain redox homeostasis and preserve physiological redox signaling. Superoxide dismutases (SODs) represent the first line of defense, catalyzing the dismutation of O_2_•^−^ into O_2_ and H_2_O_2_. Three isoforms are present in mammals: SOD1 (Cu/Zn), localized in the cytosol and mitochondrial intermembrane space; SOD2 (Mn), in the mitochondrial matrix; and extracellular SOD3 (Cu/Zn) [109,110]. H_2_O_2_ is subsequently detoxified by catalase, mainly localized in peroxisomes [111], and by glutathione peroxidases (GPx), a selenium-dependent enzyme family that reduces hydroperoxides using glutathione (GSH) as an electron donor [112]. Among non-enzymatic antioxidants, GSH is a major endogenous redox buffer, cycling between its reduced and oxidized (GSSG) forms and supporting GPx activity via glutathione reductase in an NADPH-dependent manner [113,114]. In parallel, the thioredoxin (TRX) system, together with thioredoxin reductase (TrxR) and NADPH, regulates protein redox status through disulfide bond reduction, acting as a central intracellular oxidoreductase system [115,116]. The efficiency of these antioxidant defenses declines with aging, contributing to redox imbalance. In ApoE−mice, aging is associated with increased lipid peroxidation and reduced SOD1 and SOD2 expression [117], while aged Wistar rats show decreased cardiac GSH and TRX levels [118]. In humans, aging is accompanied by reduced erythrocyte SOD activity and plasma GSH, despite a compensatory increase in catalase activity [119]. Redox homeostasis is tightly controlled by signaling pathways, among which nuclear factor erythroid 2-related factor 2 (NRF2) plays a central role by regulating antioxidant response element (ARE)-driven genes, including those involved in glutathione synthesis and the TRX system [120,121,122]. Under basal conditions, NRF2 is retained in the cytoplasm by Kelch-like ECH-associated protein 1 (KEAP1), which promotes its ubiquitination and degradation [123]. Oxidative stress disrupts KEAP1–NRF2 interaction, allowing NRF2 nuclear translocation and transcriptional activation of cytoprotective genes [124,125]. Aging is associated with increased KEAP1 expression and reduced NRF2 activity, resulting in heightened vulnerability to oxidative and endoplasmic reticulum stress [126,127]. NRF2 signaling intersects with AMPK, a key metabolic sensor activated during energetic and oxidative stress [128]. AMPK promotes NADPH homeostasis, enhances antioxidant enzyme expression (catalase, SOD2) via PGC-1α, and coordinates adaptive metabolic responses under redox stress [129,130,131,132,133]. AMPK also activates SIRT1, a NAD^+^-dependent deacetylase that regulates mitochondrial function and antioxidant defenses through deacetylation of PGC-1α and Forkhead box O (FOXO) transcription factors [134]. FOXO proteins (FOXO1, FOXO3) control the expression of key antioxidant enzymes, including SODs and GPx, and regulate autophagy, which is essential for mitochondrial quality control [135,136,137,138]. A decline in FOXO and SIRT1 activity during aging compromises antioxidant capacity and mitochondrial function [139,140,141,142,143,144,145,146]. Additional regulation is provided by SIRT6, a nuclear sirtuin involved in redox homeostasis through modulation of NADPH availability and transcriptional activation of heme oxygenase-1 (HO-1) via NRF2-dependent mechanisms (Table 2) [147,148,149].

**Figure 3:**
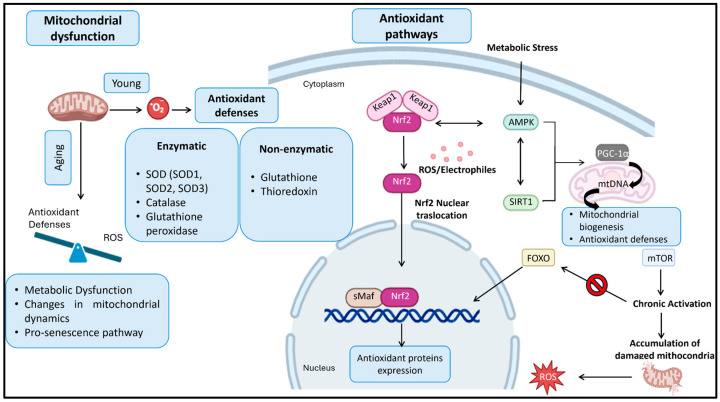
Aging is associated with mitochondrial DNA damage, impaired proteostasis, and altered mitochondrial dynamics, leading to reduced ATP production and increased ROS generation. Decreased SIRT1–PGC-1α signaling compromises mitochondrial biogenesis and antioxidant defenses, while SIRT6 supports redox homeostasis through NADPH availability and antioxidant gene expression. Chronic mTOR hyperactivation suppresses autophagy and mitophagy, promoting the accumulation of dysfunctional mitochondria, oxidative stress, and cellular senescence.

Conversely, chronic activation of mechanistic target of rapamycin (mTOR), particularly mTORC1, contributes to aging by suppressing autophagy, promoting mitochondrial dysfunction, increasing ROS levels, and inducing cellular senescence [150]. mTORC2 further limits antioxidant responses by inhibiting FOXO transcription factors [151], while excessive mTORC1 signaling favors the accumulation of dysfunctional mitochondria by reducing mitophagy despite increased mitochondrial biogenesis [152,153,154]. Collectively, the coordinated regulation of antioxidant systems and redox-sensitive signaling pathways is essential for maintaining cellular and mitochondrial homeostasis. Dysregulation of these networks during aging amplifies oxidative stress, accelerates functional decline, and highlights these pathways as key therapeutic targets in the biology of aging (Figure 3).

## 5. General Nutritional Strategies

### 5.1. Calorie Restriction and Calorie Restriction Mimetics: An Integrated Perspective on the Modulation of Aging

Nutritional modulation of aging is now considered one of the most promising strategies for extending healthspan. Recent evidence shows that diet acts not only as a source of energy, but also as a set of molecular signals capable of modulating inflammation, cellular resilience and complex interactions between the body and the microbiome [155,156,157]. In this context, specific bioactive nutrients–polyphenols, omega-3, vitamin D3—and dietary patterns such as the Mediterranean diet are key elements in steering the biological networks of aging towards more favourable trajectories [158]. Several nutritional interventions converge on this systemic view. Strategies such as targeting nutrient sensors (mTOR, AMPK, sirtuins), intermittent fasting and the Mediterranean diet act by modulating energy and metabolic inputs that profoundly influence cellular aging programmes. This approach is part of the broader “environment–food–human web”, which recognises the continuous interaction between nutritional exposures and human biological response [159].

Calorie restriction (CR) represents the most robust paradigm in the field of longevity. Several dietary patterns applied in clinical and experimental settings share energy intake reduction as a determining factor [160]. CR works by decreasing nutrient-dependent signals that activate pathways such as mTOR, improving insulin sensitivity, enhancing antioxidant processes and reducing inflammaging, with significant effects on longevity in many species [161]. The experimental support is substantial: in the rat liver model, CR reduces inflammatory markers, increases the expression of antioxidant enzymes and improves tissue architecture, confirming the intervention’s ability to counteract cell decay [162]. Alongside CR, calorie restriction mimetics (CRMs) offer a complementary approach, aimed at replicating its benefits without reducing energy intake. Molecules such as metformin, rapamycin, resveratrol and spermidine modulate key pathways such as AMPK, mTOR and SIRT1, promoting autophagy, improving mitochondrial metabolism and attenuating oxidative stress. These compounds show significant anti-aging effects on inflammation, metabolism and cell stability [163]. Some CRMs, particularly rapamycin and spermidine, also show a potential neuroprotective role, with favourable effects on cognitive decline and vulnerability to neurodegenerative disorders [164]. Overall, calorie restriction and calorie restriction mimetics represent two converging strategies for optimising the nutritional and molecular signals that govern aging. The integration of structured dietary models, precision nutrition and targeted pharmacological interventions paves the way for a more personalised and proactive approach, focused not only on lifespan but above all on maintaining a longer and better quality healthspan.

### 5.2. Intermittent Fasting and Time-Restricted Feeding

Intermittent fasting (IF) and time-restricted eating (TRE) offer accessible nutritional strategies to counteract age-related metabolic and cognitive decline, with robust evidence emerging from recent human randomized controlled trials. Large-scale network meta-analyses of dozens of RCTs in obese adults demonstrate that IF protocols—such as alternate-day fasting, 5:2 regimens, and TRE—achieve weight loss comparable to continuous calorie restriction, though long-term adherence remains challenging, particularly for more restrictive approaches [165]. Building on these findings, mechanistic studies reveal that short-term TRE enhances systemic autophagy in healthy adults, especially in skeletal muscle, via cyclic activation of AMPK/mTOR and NAD+/SIRT1 pathways—processes pivotal to proteostasis and senescence delay, with stronger effects in those over age 50 [166]. Furthermore, systematic reviews confirm TRE improves memory and processing speed in mild cognitive impairment, reduces systemic inflammation, and boosts neuroprotective BDNF, with moderate 10–14 h eating windows proving most tolerable in older populations [167]. Umbrella reviews of meta-analyses affirm IF’s benefits on weight, glycemic control, and lipids—particularly in obesity and type 2 diabetes—while TRE matches calorie restriction without enforced deficits, though direct longevity data quality remains limited [168].

Taken together, these converging lines of evidence position IF/TRE as promising adjuncts to standard care for healthy aging. Ultimately, TRE suppresses key biomarkers of aging such as IGF-1 and mTOR while preserving lean mass in older adults, but long-term adherence is lacking, which points to the need for tailored multicentre studies focused on clinical outcomes such as frailty and survival [169]. Such efforts will clarify optimal protocols and patient selection to maximise translational impact.

### 5.3. Anti-Inflammatory Diets (Mediterranean, Plant-Based, DASH): Impact on ROS, Inflammation and Mitochondria

Aging is determined by three interconnected factors: oxidative stress caused by ROS, low-grade chronic inflammation and mitochondrial dysfunction leading to bioenergetic failure and cellular senescence [170]. Plant-rich dietary patterns, namely the Mediterranean diet (MD), plant-based diets (PBDs) and the DASH diet, counteract these processes through antioxidants, polyphenols, fibre and anti-inflammatory nutrients that modulate Nrf2 signalling, the NLRP3 inflammasome, PGC-1α mitophagy and epigenetic clocks [171]. Dietary fibers exert indirect but highly relevant antioxidant and anti-inflammatory effects through their interaction with the gut microbiota. Indeed, unlike classical antioxidants, fibers do not directly scavenge reactive oxygen species; instead, they modulate redox homeostasis by shaping microbial metabolism and host signaling pathways.

Regarding the polyphenols, they act primarily as modulators of redox-sensitive signaling pathways rather than as direct radical scavengers. These compounds activate the NRF2–KEAP1 axis, promoting the transcription of antioxidant and detoxifying enzymes, including heme oxygenase-1 (HO-1), glutathione S-transferases, and NAD(P)H quinone oxidoreductase 1 (NQO1) [171]. In parallel, polyphenols inhibit pro-inflammatory signaling cascades, notably NF-κB and MAPK pathways, thereby reducing cytokine production and limiting ROS amplification during chronic inflammation.

The MD, characterised by high consumption of fruit, vegetables, olive oil and fish, exerts powerful anti-inflammatory effects that mitigate age-related decline by modulating ROS, low-grade chronic inflammation (inflammaging) and mitochondrial dysfunction [172]. Growing evidence from recent high-impact studies shows that adherence to the MD reduces markers of oxidative stress and preserves mitochondrial integrity, key factors in cellular senescence and longevity [173]. MD polyphenols, such as hydroxytyrosol in extra virgin olive oil, directly eliminate ROS and inhibit NLRP3 inflammasome activation, thereby attenuating mitochondrial ROS leakage and mtDNA damage observed in aging [174,175]. Clinical interventions confirm dose-dependent reductions in systemic ROS (e.g., 8-OHdG) and inflammatory cytokines (IL-6, TNF-α) after MD, with partial mediation via Nrf2/upregulated antioxidant pathways and PGC-1α-mediated mitophagy [176,177]. In cohorts of pregnant women, an indicator of accelerated aging stress, the MD reduced ROS by 25–30% while increasing total antioxidant capacity (TAC), highlighting replicable effects throughout life [178].

Mitochondrial bioenergetic benefits are profound: the MD improves electron transport chain efficiency and reduces proton loss, counteracting age-induced fission/fusion imbalance [179]. Longitudinal data from the UK Biobank link high MD scores to a slowing of the epigenetic clock (PhenoAge), with inflammation and ROS accounting for 15–20% of the variance [180]. 

PBDs, rich in fruit, vegetables, legumes and whole grains, exert powerful anti-inflammatory effects that counteract age-related decline by suppressing ROS, inflammaging and mitochondrial deterioration [181]. Recent high-impact systematic reviews and RCTs demonstrate that adherence to a vegan/vegetarian diet reduces biomarkers of oxidative stress (MDA, 8-OHdG) while improving mitochondrial biogenesis via PGC-1α, positioning PBDs as viable alternatives to Mediterranean models for longevity [182,183]. PBD polyphenols and fibre-derived metabolites directly extinguish ROS and attenuate NLRP3/IL-1β signalling, mitigating the mtROS overload and oxidative mtDNA damage that characterise senescence [184]. Interventions lasting 6–24 months produce dose-dependent decreases in IL-6, TNF-α and CRP, mediated by Nrf2-driven antioxidants and upregulated mitophagy [185,186]. In proxies of metabolic syndrome for accelerated aging, vegan PBDs reduce ROS/MCP-1 inflammation by 20–30% along with increased β-oxidation capacity [187].

Mitochondrial dynamics also improve markedly as PBDs restore electron transport chain coupling, curb proton leakage, and balance Drp1/Mfn2 fission-fusion under oxidative stress [182]. A study from the UK Biobank links high PBD scores to a slowdown in epigenetic aging, with ROS/inflammation mediating a reduction in the acceleration of phenotypic age effects [188]. Such evidence supports PBDs as scalable geroprotectors, synergising with exercise in clinical trials to extend healthy lifespan.

The dietary approaches to stop hypertension diet (DASH), rich in fruit, vegetables, low-fat dairy products, whole grains and low in sodium, has powerful anti-inflammatory and antioxidant effects that mitigate ROS production, inflammation and mitochondrial stress in aging [189,190]. Meta-analyses of RCTs confirm that the DASH diet significantly reduces hs-CRP (−1.01 mg/L compared to the usual diet) and oxidative markers (MDA SMD −0.53, GSH SMD +0.83), with greater effects in studies lasting ≥8 weeks [189]. These benefits position the DASH diet as an accessible model for healthy aging, comparable to MDs/PBDs in suppressing age-related molecular damage.

MDs, PBDs, and DASH converge on shared mechanisms—ROS elimination, inflammasome inhibition, and mitochondrial rescue—that produce substantial reductions in aging biomarkers in RCTs and meta-analyses [191]. These models offer precision nutritional synergies with senolytics, metformin, or exercise, warranting multicentre studies to extend healthy lifespan in frail populations. 

## 6. Key Micronutrients: Vitamins, Antioxidants

Micronutrients play a fundamental role in preserving cellular homeostasis and regulating multiple biological processes involved in aging. Vitamins and trace elements contribute both directly and indirectly to the maintenance of redox balance, mitochondrial function, genomic stability, and adaptive responses to physiological stress. With advancing age, progressive dysregulation of these systems promotes mitochondrial dysfunction, increased oxidative stress, chronic low-grade inflammation (inflammaging), reduced metabolic flexibility, and a gradual loss of biological resilience, all of which contribute to age-related functional decline [192].

From an aging perspective, these alterations are particularly relevant because the capacity to respond effectively to metabolic and environmental stressors progressively deteriorates over time. Age-associated impairments in redox buffering, immune regulation, and stress-response signaling amplify the biological consequences of micronutrient inadequacy, accelerating cellular dysfunction and increasing susceptibility to age-related diseases [193].

Historically, antioxidant vitamins have been primarily regarded as scavengers of ROS, protecting lipids, proteins, and DNA from oxidative damage. However, contemporary evidence has challenged this reductionist interpretation, demonstrating that ROS are not merely harmful metabolic by-products but also function as tightly regulated signaling molecules required for adaptive cellular responses, including mitochondrial biogenesis and metabolic remodeling [194].

Accordingly, micronutrients should be conceptualized as modulators of a dynamic equilibrium between ROS generation and endogenous defense mechanisms rather than as simple antioxidant agents. Vitamins such as vitamin C, vitamin E, and carotenoids, together with trace elements including selenium, zinc, copper, and manganese, are essential for the optimal function of enzymatic antioxidant systems, acting as cofactors for key enzymes such as superoxide dismutase, glutathione peroxidase, and catalase.

At the molecular level, redox status and micronutrient availability also influence telomere dynamics. Excessive ROS exposure has been associated with accelerated telomere shortening and reduced telomerase activity, thereby promoting premature replicative senescence. These mechanisms are particularly relevant in aging, as telomere attrition represents not only a biomarker but also a driver of cellular senescence and tissue dysfunction [193].

Given that cognitive decline represents a key hallmark of aging, vitamins emerge as important anti-aging factors due to their potential to mitigate both physiological, age-related cognitive decline and the decline associated with pathological conditions, including neurodegenerative diseases. In this context, a healthy lifestyle has been consistently associated with a slower progression of dementia. However, it is important to consider that aging is accompanied by a reduced capacity to absorb certain micronutrients, which may compromise adequate nutritional status. Consequently, dietary supplementation may be necessary to maintain sufficient micronutrient levels. Moreover, age-related lifestyle changes—such as reduced physical activity and decreased exposure to sunlight—can further limit the intake and bioavailability of specific vitamins, including vitamin D, reinforcing the relevance of targeted supplementation strategies in older individuals [195].

It is well established that B-group vitamins play a fundamental role in cognitive functions, particularly in memory processes. In addition, deficiencies in vitamin B12 have been associated with the onset or accelerated worsening of several hallmarks of aging, including increased DNA damage and mitochondrial dysfunction. Furthermore, vitamin B12 deficiency has been shown to promote a pro-inflammatory state, characterized by enhanced release of pro-inflammatory interleukins [196]. 

Many of the anti-aging roles attributed to micronutrients are closely associated with their antioxidant properties, which contribute to the maintenance of redox homeostasis and the prevention of age-related cellular damage [197].

In particular, several vitamins contribute to the maintenance of redox homeostasis either by directly scavenging reactive oxygen and nitrogen species or by supporting endogenous antioxidant defense systems. Vitamin C and vitamin E are among the most extensively studied antioxidant vitamins and act synergistically to protect cellular components from oxidative damage. Vitamin C functions as a potent water-soluble antioxidant, neutralizing free radicals and regenerating oxidized vitamin E, thereby preserving membrane integrity and preventing lipid peroxidation [198]. Vitamin E, in turn, plays a critical role in protecting polyunsaturated fatty acids within biological membranes from oxidative stress, a process that is particularly relevant in aging tissues characterized by increased lipid peroxidation and mitochondrial dysfunction. Through its antioxidant function, vitamin E has been shown to contribute to the prevention of dementia, not only during physiological aging but also under pathological conditions such as neurodegenerative diseases. In particular, in Alzheimer’s disease, vitamin E prevents the formation of amyloid-β–associated reactive oxygen species. Moreover, vitamin E activates protein phosphatase 2A (PP2A), a phosphatase involved in tau homeostasis that is reduced in the brains of patients with Alzheimer’s disease [199,200].

Beyond their direct antioxidant activity, several vitamins exert anti-aging effects through indirect modulation of oxidative stress, inflammation, and mitochondrial function. B-group vitamins, particularly vitamin B12 and folate, are essential for one-carbon metabolism and methylation reactions, and their deficiency has been associated with increased oxidative stress, genomic instability, and mitochondrial dysfunction—key hallmarks of aging [201].

In this context, vitamins and micronutrients may also exert downstream effects, for example by supporting the maintenance of mitochondrial integrity and function. Mitochondrial network integrity further depends on the efficiency of mitophagy, a selective autophagic process responsible for the removal of damaged mitochondria. Impaired mitophagy leads to the accumulation of dysfunctional organelles and sustained ROS overproduction, accelerating age-associated functional decline [202].

Micronutrients play a pivotal role in this scenario by acting as essential cofactors that sustain core mitochondrial metabolic functions, including ATP production, heme biosynthesis, assembly and stability of electron transport chain complexes, and the detoxification of ROS. Insufficient availability of specific micronutrients results in mitochondrial dysfunction, characterized by impaired bioenergetics, increased oxidative stress, and altered mitochondrial dynamics. These alterations ultimately compromise mitochondrial quality control mechanisms, including mitophagy, leading to the accumulation of dysfunctional mitochondria [203]. By preserving mitochondrial integrity and supporting efficient mitochondrial turnover, adequate micronutrient availability contributes to the maintenance of cellular homeostasis and resilience during aging and in age-related pathologies.

Despite the essential role of micronutrients in cellular protection, the literature highlights important limitations associated with indiscriminate antioxidant supplementation. Within the framework of anti-aging strategies, it is important to recognize that controlled stress leading to ROS production is not invariably harmful but may instead confer beneficial effects. In this regard, physical exercise is widely regarded as a cornerstone of a healthy lifestyle that promotes longevity. Exercise induces a mitohormetic response through transient increases in mitochondrial ROS, which act as signaling molecules to activate adaptive cellular and systemic pathways, ultimately enhancing stress resistance, mitochondrial biogenesis, and metabolic flexibility [204]. In this context, micronutrients play a complementary and essential role by supporting mitochondrial metabolism and redox homeostasis. Adequate availability of vitamins and micronutrients ensures efficient antioxidant defenses and mitochondrial quality control, allowing cells to appropriately respond to hormetic stimuli without tipping toward pathological oxidative stress. Together, mitohormesis induced by physical activity and micronutrient-dependent mitochondrial support converge to preserve mitochondrial integrity, promote efficient turnover of damaged organelles, and counteract key hallmarks of aging. For this reason, chronic high-dose supplementation with exogenous antioxidants may blunt physiological redox signaling and attenuate adaptive responses [205].

Indeed, it has been demonstrated that long-term supplementation with antioxidant vitamins, such as vitamins C and E, may negatively impact lifespan [206]. These findings have contributed to a re-evaluation of the role of ROS in aging and have fueled the debate surrounding the classical theories of aging. The traditional free radical theory of aging, originally proposed by Harman, postulates that aging results primarily from the cumulative damage inflicted by ROS on cellular macromolecules, including DNA, proteins, and lipids [207]. According to this view, oxidative stress represents a central driver of most aging hallmarks. However, other revised models of aging emerged. In particular, the mitohormesis concept suggests that low and transient increases in ROS act as signaling molecules that trigger adaptive stress responses, ultimately promoting longevity and metabolic health rather than accelerating aging. Consequently, contemporary views of aging emphasize the importance of redox balance, mitochondrial quality control, and adaptive stress responses, rather than indiscriminate suppression of oxidative stress, as key determinants of healthy aging and longevity.

A similar requirement for fine regulation applies to trace elements. Trace micronutrients, including essential minerals such as zinc, selenium, iron, copper, and manganese, play crucial roles in maintaining cellular homeostasis and metabolic integrity throughout the lifespan. These elements act as indispensable cofactors for a wide range of enzymes involved in antioxidant defense, mitochondrial respiration, DNA repair, and immune regulation. During aging, alterations in trace element homeostasis are frequently observed and have been associated with increased oxidative stress, mitochondrial dysfunction, immune senescence, and chronic low-grade inflammation [208]. In particular, deficiencies or imbalances in zinc and selenium have been linked to impaired antioxidant enzyme activity, including superoxide dismutases and glutathione peroxidases, thereby exacerbating oxidative damage and contributing to age-related functional decline. In particular, zinc and selenium status has been linked to multimorbidity in individuals over 60 years of age, with evidence indicating that most older adults fail to meet the recommended daily intake of these micronutrients [209]. Indeed, selenium is essential for selenoprotein activity, whereas both deficiency and excess intake have been associated with adverse outcomes in aging populations [210].

Overall, aging should be interpreted as a progressive loss of biological adaptability rather than a simple accumulation of molecular damage. Within this framework, key micronutrients emerge as regulators of stress-adaptive processes that govern cellular maintenance, inflammatory balance, and metabolic resilience throughout the aging trajectory.

## 7. Bioactive and Nutraceuticals

**Figure 4:**
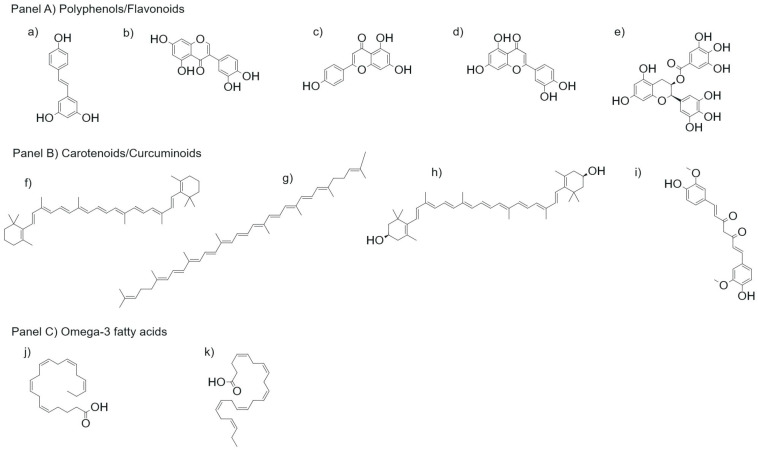
Representative chemical structures of major nutraceutical classes involved in antioxidant and redox-modulating activity. Panel (**A**): Polyphenols and Flavonoids (a) resveratrol, (b) quercetin, (c) apigenin, (d) luteolin, (e) epigallocatechin gallate. Panel (**B**): Carotenoids and Curcuminoids (f) β-carotene, (g) lycopene, (h) lutein, (i) curcumin. Panel (**C**): Omega-3 fatty acids (j) eicosapentaenoic acid, EPA; (k) docosahexaenoic acid, DHA.

A growing body of research suggests that bioactive dietary components, although not essential nutrients, play a critical role in modulating molecular pathways involved in aging, including oxidative stress, chronic low-grade inflammation, mitochondrial function, and epigenetic regulation. Among these, polyphenols, carotenoids, curcuminoids, organosulfur compounds, omega-3 fatty acids, and microbiota-derived metabolites have emerged as key modulators of healthspan. This section synthesizes current evidence on their mechanisms of action and therapeutic potential within the context of nutritional strategies for healthy aging. Representative chemical structures of the main classes of nutraceuticals with antioxidant activity discussed in Section 7.1, Section 7.2 and Section 7.3 are shown in Figure 4.

### 7.1. Polyphenols: Resveratrol, Flavonoids, and Catechins

Resveratrol, a naturally occurring stilbene abundant in grapes, berries, and peanuts, is one of the most extensively studied polyphenols for its role as a caloric restriction mimetic. Its biological activity is primarily mediated by activation of the SIRT1/AMPK–PGC-1α signaling axis, promoting mitochondrial biogenesis, metabolic flexibility, and energy homeostasis. SIRT1 activation induces PGC-1α deacetylation, enhancing mitochondrial DNA replication and the transcription of genes involved in oxidative phosphorylation and fatty acid oxidation, while AMPK activation reinforces insulin sensitivity and glucose and lipid metabolism, which decline with aging [211]. At the mitochondrial level, resveratrol improves electron transport chain efficiency by increasing the activity of complexes I and IV, thereby limiting electron leakage and mitochondrial ROS production, and induces endogenous antioxidant enzymes, including SOD, catalase, and glutathione peroxidase, protecting mitochondrial membranes and mtDNA [212]. Beyond redox regulation, resveratrol acts as an epigenetic modulator via SIRT1-dependent histone deacetylation, reducing pro-inflammatory gene expression and promoting antioxidant and DNA-repair pathways. It also modulates microRNA (miRNA) networks involved in metabolic homeostasis and stress resistance, including upregulation of miR-21 and miR-126 and suppression of inflammatory miR-155 and senescence-associated miR-34a [211]. Emerging evidence further supports a gut–mitochondria axis, whereby resveratrol reshapes gut microbiota composition, favoring butyrate-producing bacteria such as *Faecalibacterium prausnitzii* and *Roseburia*, whose short-chain fatty acids (SCFAs) enhance mitochondrial respiration and anti-inflammatory signaling [213]. Despite strong mechanistic support, clinical outcomes remain variable due to limited oral bioavailability, rapid hepatic metabolism, and interindividual differences in microbiota-dependent conversion to active metabolites such as dihydroresveratrol and lunularin.

Flavonoids (e.g., quercetin, apigenin, luteolin) and catechins, notably epigallocatechin gallate (EGCG), exert pleiotropic anti-aging effects primarily through hormetic activation of redox-sensitive signaling pathways rather than direct ROS scavenging. A central mechanism involves activation of the Nrf2-Keap1 pathway, leading to Nrf2 nuclear translocation and ARE-driven transcription of phase II detoxification enzymes, including HO-1, glutathione S-transferase (GST), and NAD(P)H quinone oxidoreductase-1 (NQO1), thereby strengthening endogenous antioxidant defenses [214]. These compounds also modulate mitochondrial quality control by promoting PINK1/Parkin-dependent mitophagy and mitochondrial biogenesis via upregulation of PGC-1α and TFAM, preserving mitochondrial integrity and ATP production in neuronal and skeletal muscle models [215]. In parallel, flavonoids and catechins suppress chronic inflammation by inhibiting NF-κB and MAPK signaling, reducing IL-6, TNF-α, and IL-1β expression, and attenuating NLRP3 inflammasome activation [216].

Epigenetic regulation represents an additional layer of action, as flavonoids such as EGCG, genistein, and luteolin inhibit DNA methyltransferases (DNMTs) and HDACs, leading to selective reactivation of tumor-suppressor and longevity-associated genes. They also regulate non-coding RNAs involved in aging-related inflammation and senescence, including downregulation of miR-146a and miR-34a [217]. Consistent with these molecular effects, clinical and translational studies indicate that chronic intake of flavonoid- and catechin-rich foods or standardized extracts improves vascular function, insulin sensitivity, and lipid metabolism. Randomized controlled trials report that supplementation with quercetin (500 mg/day) or green tea catechins (400–800 mg/day) reduces fasting glucose, triglycerides, and inflammatory biomarkers in aging populations [218]. However, efficacy is dose-dependent and influenced by interindividual variability in gut microbiota composition, hepatic conjugation, and microbiome-driven conversion into bioactive metabolites such as valerolactones and phenyl-γ-valerolactones.

Overall, polyphenols including resveratrol, flavonoids, and catechins coordinately modulate nutrient-sensing, antioxidant, inflammatory, and epigenetic networks underlying cellular aging. By targeting SIRT1/AMPK, Nrf2/PGC-1α, and NF-κB/NLRP3 axes, they enhance mitochondrial quality control, stabilize the epigenome, and improve metabolic resilience. Although translation to consistent clinical benefits requires optimized bioavailability and personalized nutrition strategies, these compounds represent key nutraceutical tools for promoting healthy longevity.

### 7.2. Carotenoids, Curcuminoids, and Other Phytochemicals

Within the framework of nutritional strategies for healthy aging, carotenoids, curcuminoids, and other phytochemicals emerge as central dietary components capable of influencing the molecular networks that drive longevity and resilience. Unlike essential micronutrients, these compounds act as metabolic modulators and redox sensors, fine-tuning signaling cascades related to oxidative stress, inflammation, mitochondrial function, and gene expression. Their pleiotropic biological activity positions them as pivotal elements of a dietary pattern aimed not only at disease prevention but also at extending healthspan through the maintenance of systemic homeostasis.

Carotenoids, a class of lipid-soluble pigments including β-carotene, lutein, zeaxanthin, and lycopene, display potent antioxidant and anti-inflammatory properties. These molecules are integrated into cellular and mitochondrial membranes where they preserve lipid integrity, prevent peroxidation, and stabilize redox homeostasis. Mechanistically, carotenoids function as *singlet oxygen quenchers* and modulate intracellular signaling through activation of Nrf2 and suppression of NF-κB and IL-6, thus attenuating chronic low-grade inflammation typical of aging. In human studies, long-term dietary carotenoid intake correlates with decreased circulating levels of oxidative stress markers such as malondialdehyde and 8-OHdG, alongside improved total antioxidant capacity, supporting their role as biomarkers and mediators of slowed biological aging [219,220]. Experimental evidence also suggests that carotenoids enhance mitochondrial efficiency and prevent depolarization by preserving cardiolipin stability and reducing ROS leakage, contributing to improved metabolic flexibility during aging.

Among polyphenolic phytochemicals, curcuminoids, especially *curcumin*, the principal bioactive from *Curcuma longa*, exhibit a broad spectrum of *geroprotective* actions. Curcumin simultaneously activates Nrf2, AMPK/SIRT1, and PGC-1α signaling pathways, enhancing antioxidant defenses, promoting mitochondrial biogenesis, and suppressing *inflammaging* via NF-κB and NLRP3 inflammasome inhibition [221]. Moreover, curcumin modulates epigenetic mechanisms—including inhibition of HATs and DNMTs—leading to transcriptional reactivation of antioxidant and anti-apoptotic genes while repressing pro-inflammatory loci. Its neuroprotective activity is well documented: curcumin upregulates brain-derived neurotrophic factor (BDNF), enhances synaptic plasticity, and reduces β-amyloid aggregation, mitigating cognitive decline in aging models [222]. Clinical trials have demonstrated that sustained curcumin supplementation (≥500 mg/day for 12 weeks) reduces systemic oxidative stress and inflammatory cytokines while improving endothelial function, highlighting its translational potential as a dietary adjuvant in age-related metabolic dysfunction.

Beyond these key classes, other bioactive phytochemicals, such as sulforaphane from cruciferous vegetables and allicin and organosulfur compounds from garlic and onions, act through *xenohormetic signaling* to reinforce antioxidant defenses and mitochondrial quality control. Sulforaphane potently induces Nrf2-dependent transcription of phase II detoxification enzymes, whereas allicin exerts vascular-protective effects by reducing endothelial ROS and modulating MAPK/NF-κB signaling. Organosulfur compounds also display epigenetic plasticity, modulating microRNAs (e.g., miR-21, miR-200c) and enhancing SIRT1/NQO1 expression, further integrating redox metabolism with genomic stability.

Collectively, these phytochemicals converge on shared nutrient-sensing and stress-response networks, Nrf2/SIRT1/PGC-1α activation and NF-κB/NLRP3 inhibition, that underpin their ability to slow functional decline, sustain mitochondrial competence, and preserve metabolic flexibility. Their inclusion within dietary frameworks such as the Mediterranean and plant-based diets provides a synergistic platform for promoting *healthy aging* through sustained modulation of oxidative, inflammatory, and epigenetic pathways. As future research advances toward precision nutrition, integrating *nutrigenomic profiling* and *bioavailability-optimized formulations*, carotenoids, curcuminoids, and related phytochemicals are poised to become key agents in nutritional strategies designed to extend healthspan and delay the onset of age-associated pathophysiology.

### 7.3. Omega-3 Fatty Acids: Anti-Inflammatory Actions and Membrane Dynamics

Omega-3 long-chain polyunsaturated fatty acids (n-3 LC-PUFAs), particularly eicosapentaenoic acid (EPA; 20:5n-3) and docosahexaenoic acid (DHA; 22:6n-3), are critical bioactive lipids incorporated into cellular phospholipid membranes throughout the body. Beyond their classical roles in lipid metabolism, these fatty acids are potent modulators of inflammation, membrane biophysics, and gene expression. In the context of aging, the physiological significance of omega-3 fatty acids lies in their ability to counteract “inflammaging”, a state of chronic, low-grade inflammation that accelerates tissue degeneration and metabolic dysfunction. Aging is characterized by an imbalance between pro-inflammatory cytokines (TNF-α, IL-6, IL-1β) and the body’s capacity to resolve inflammation. Omega-3 PUFAs restore this equilibrium by generating specialized pro-resolving mediators (SPMs) and by reshaping cellular membrane architecture, leading to improved signaling fidelity and stress resilience [223].

EPA and DHA compete with arachidonic acid (AA, 20:4n-6) for the cyclooxygenase (COX) and lipoxygenase (LOX) enzyme systems. This competition reduces the formation of potent pro-inflammatory eicosanoids such as prostaglandin E_2_ (PGE_2_) and leukotriene B_4_ (LTB_4_), while favoring the synthesis of less inflammatory counterparts, including PGE_3_ and LTB_5_. Moreover, COX-2 can catalyze the formation of electrophilic oxo-derivatives (EFOX) from DHA and docosapentaenoic acid (DPA). These reactive lipid species covalently modify cysteine residues on transcriptional regulators such as Keap1, activating Nrf2-dependent antioxidant gene networks, which upregulate HO-1 and GST. This dual anti-inflammatory and antioxidant mechanism reduces NF-κB activation and cytokine expression [224]. Moreover, SPMs—including resolvins (RvE1, RvD1), protectins (PD1), and maresins (MaR1)—are biosynthesized from EPA and DHA through enzymatic conversion by 15-LOX and COX-2. These molecules promote the active resolution phase of inflammation, enhancing macrophage phagocytosis of apoptotic cells and inhibiting neutrophil infiltration. SPMs bind to G-protein–coupled receptors such as ChemR23 (for RvE1) and ALX/FPR2 (for RvD1), triggering intracellular signaling that downregulates nuclear translocation of NF-κB and AP-1, reduces IL-1β, IL-6, and TNF-α transcription, and enhances IL-10 expression. This reprogramming of the immune milieu shifts macrophages toward an anti-inflammatory M2 phenotype, promoting tissue homeostasis [223].

EPA and DHA serve as endogenous ligands for GPR120 (also known as FFAR4), a membrane receptor highly expressed in adipose tissue, macrophages, and skeletal muscle. Upon activation, GPR120 recruits β-arrestin 2, leading to internalization of the receptor–TLR4 complex and subsequent inhibition of NLRP3 inflammasome activation. This pathway attenuates IL-1β release and improves insulin sensitivity, contributing to anti-inflammatory and metabolic benefits particularly relevant in age-associated metabolic syndrome [225]. Moreover, emerging evidence indicates that omega-3 fatty acids can alter DNA methylation (DNAm) patterns in genes governing inflammatory signaling, such as TLR4, IL6, and TNFA. In a six-month intervention study, dietary n-3 PUFA supplementation induced promoter hypermethylation in pro-inflammatory genes and reduced expression of key cytokines in peripheral blood mononuclear cells, highlighting an epigenetic mechanism contributing to the anti-inflammatory phenotype [226].

During the aging process, cellular membranes undergo progressive compositional alterations characterized by a decline in DHA content, an increase in saturated fatty acids, and elevated levels of lipid peroxidation. These biochemical changes reduce membrane fluidity and deformability, impairing the lateral mobility of integral proteins and leading to reduced receptor sensitivity and defective signal transduction.

Dietary or supplemental omega-3 fatty acids can restore phospholipid asymmetry, enhance membrane elasticity, and normalize the distribution of polyunsaturated acyl chains between membrane leaflets. This remodeling improves receptor–ligand interactions, optimizes ion channel kinetics, and supports synaptic vesicle fusion and neurotransmitter release. These effects are particularly significant in neuronal and immune cells, where efficient membrane signaling is crucial for maintaining cognitive and immunological function.

Furthermore, DHA serves as a precursor to protectins (PD1) and neuroprotectins (NPD1), lipid mediators that bolster neuronal survival under oxidative and excitotoxic stress. These mediators stabilize mitochondrial membranes, limit calcium influx, and preserve synaptic microdomain organization essential for synaptic plasticity and long-term potentiation (LTP). Collectively, these actions establish a neuroprotective link between membrane dynamics and healthy aging, underscoring the central role of omega-3 fatty acids in mitigating age-related cognitive and cellular decline [223].

At the systemic level, a higher red blood cell (RBC) omega-3 index—defined as the combined percentage of EPA and DHA in total RBC fatty acids—has been consistently associated with lower circulating concentrations of inflammatory biomarkers, including C-reactive protein (CRP), IL-6, and tumor necrosis factor receptor-2 (TNFR2). These inverse correlations indicate that enhanced tissue incorporation of omega-3 fatty acids translates into reduced systemic inflammation and improved vascular and metabolic homeostasis [227]. In aging populations, chronic omega-3 intake exerts multiple protective physiological effects. These include decreased expression of endothelial adhesion molecules (VCAM-1, ICAM-1, E-selectin), which reduces leukocyte adhesion and endothelial activation; improved arterial compliance and endothelial nitric oxide availability, which enhance vascular elasticity; and augmented mitochondrial bioenergetics, promoting ATP production and reducing oxidative burden in skeletal and cardiac muscle.

The combined impact of these mechanisms mitigates the progression of atherosclerosis, sarcopenia, cognitive decline, and metabolic syndrome, all of which share chronic inflammation and oxidative stress as underlying pathophysiological drivers. Thus, omega-3 fatty acids emerge as key nutritional modulators of the aging phenotype, acting simultaneously on cellular membranes, inflammatory networks, and metabolic resilience to preserve systemic health and functional longevity.

### 7.4. Probiotics and Postbiotics: Modulators of Systemic Inflammation and Oxidative Metabolism

Probiotics and their postbiotic derivatives emerge as compelling nutritional modulators of systemic inflammation and oxidative metabolism, processes intrinsically linked to aging and age-related diseases via chronic low-grade inflammation and redox imbalance. Aging is associated with gut microbiota dysbiosis, increased intestinal permeability, and elevated systemic endotoxin exposure, all of which amplify pro-inflammatory signaling and oxidative stress; targeted interventions aimed at gut microbiota modulation therefore offer promising strategies to attenuate these maladaptive responses (e.g., gut-microbiome aging relationship) [228]. Mechanistically, probiotics such as Lactobacillus and Bifidobacterium strains can restore microbial equilibrium, reduce intestinal permeability, lower lipopolysaccharide (LPS) translocation into circulation, and stimulate production of short-chain fatty acids (SCFAs), notably acetate, propionate, and butyrate, that engage host immune and metabolic pathways to mitigate NF-κB–mediated inflammation and support epithelial barrier integrity, thereby reducing systemic cytokines like TNF-α and CRP in metabolic disease contexts [229]. Meta-research aggregating multiple randomized controlled trials indicates that probiotic supplementation can significantly reduce biomarkers of inflammation (CRP, TNF-α) and oxidative stress (MDA) while enhancing antioxidant defenses (GSH, TCA), although effects on IL-6 show variability potentially attributable to strain specificity and intervention heterogeneity [229]. In older adults specifically, probiotic intervention has demonstrated feasibility in attenuating inflammatory phenotypes associated with aging, though evidence remains limited and heterogeneous in healthy elderly populations, underscoring the need for well-powered, strain-specific trials in this demographic [230]. 

Clinical research investigating the impact of probiotic supplementation on systemic inflammation and oxidative metabolism. A double-blind, placebo-controlled RCT in older adults examined the effect of *Lactiplantibacillus plantarum* HEAL9 supplementation alone or combined with berry polyphenols on biomarkers of inflammaging, intestinal barrier integrity, and oxidative stress. Although full biomarker results for IL-6, TNF-α, and CRP were mixed due to variability in response and study power, trends toward reduced pro-inflammatory signaling and improved gut microbial profiles were reported, supporting the concept that targeted probiotic strains can influence systemic immunometabolic status in aging individuals [231].

Beyond age-specific cohorts, broader meta-analytic evidence from heterogeneous adult populations—including metabolic disease, neurological disorders, and non-communicable conditions—demonstrates that probiotic supplementation can significantly reduce inflammatory markers such as CRP and TNF-α and lower oxidative stress markers such as MDA, while enhancing endogenous antioxidant defenses like total GSH. For example, in a 2025 systematic review and meta-analysis of RCTs, probiotic supplementation was associated with significant decreases in CRP (standardized mean difference [SMD] ≈ −1.33) and TNF-α (SMD ≈ −1.10), as well as reductions in MDA (SMD ≈ −1.38) and increases in GSH (SMD ≈ +0.65) compared to placebo or control groups across diverse adult populations with non-communicable diseases. However, effects on IL-6, NO, and TAC were less consistent, highlighting variability attributable to probiotic strains, dosages, intervention durations, and baseline health status [232].

Specific clinical trials further illustrate biomarker shifts after probiotic interventions. In adults with Alzheimer’s disease or mild cognitive impairment (MCI)—conditions where inflammaging and oxidative stress are central—randomized controlled trials using multi-strain *Lactobacillus* and *Bifidobacterium* formulations over 12-week periods reported significant reductions in malondialdehyde and high-sensitivity CRP levels, alongside improvements in cognitive performance measures, suggesting that modulation of systemic inflammation and oxidative imbalance may contribute to these effects. The meta-analysis of these trials found SMD ≈ −0.60 for MDA and SMD ≈ −0.57 for high-sensitivity CRP in the probiotic group versus controls [233].

Across clinical settings, whether cardiometabolic, neurological, or immune-related, probiotic supplementation has repeatedly shown biologically relevant reductions in inflammation and oxidative stress biomarkers, albeit with heterogeneity in magnitude and consistency. Meta-analytic evidence further reinforces that probiotic interventions reduce hs-CRP and MDA levels and improve antioxidant status even when applied to chronic disease populations, a finding relevant to aging given the shared pathways of inflammaging and oxidative burden [234].

Mechanistically, these clinical effects are thought to arise from probiotics’ capacity to *restore gut microbiota balance*, *strengthen epithelial barrier integrity*, *reduce systemic endotoxin (LPS) translocation*, and *stimulate production of beneficial metabolites such as short-chain fatty acids (SCFAs)* that modulate immune cell function—ultimately leading to dampened NF-κB–mediated inflammatory signaling and enhanced redox homeostasis. Although strain-specific bioactivity, optimal dosing, and long-term efficacy in aging populations remain areas needing clarification, current clinical evidence supports the concept that probiotics act on systemic inflammatory and oxidative pathways relevant to age-related physiological decline. However, heterogeneity in study design, strain specificity, and intervention duration underscores the need for large-scale, long-duration RCTs specifically in older populations to establish probiotics as a validated nutritional strategy against inflammaging and oxidative stress.

## 8. Food Technologies and Bioavailability 

### 8.1. Influence of Food Processing on Bioactive Molecules

Food processing technologies have a significant impact on the stability and concentration of bioactive compounds with antioxidant activity [235,236,237]. Conventional heat treatments, such as pasteurisation and sterilisation, can cause the degradation of heat-labile molecules such as water-soluble vitamins and polyphenols [238,239]. Vitamin C, for example, suffers losses of between 15% and 55% during high-temperature thermal processes, with degradation kinetics depending on pH and temperature conditions [240]. Omega-3 fatty acids, containing double bonds, are highly susceptible to oxidation when exposed to processes involving contact with air, heat, light and transition metals [241].

In contrast, some innovative technologies have proven to be more conservative with regard to bioactive phytochemicals. High hydrostatic pressure treatments, typically operating at pressures of 400–600 MPa at room or moderate temperatures, allow microorganisms to be inactivated while preserving the molecular structure of antioxidant compounds [242]. Comparative studies have shown that the anthocyanin content in fruit juices treated with high pressure is 20–40% higher than in thermally pasteurised juices demonstrated 7–12% increases in lycopene content in tomato juice treated with combined pressure-heat techniques [243,244,245].

Extraction technologies are another critical aspect for preserving antioxidant activity. Extraction with supercritical fluids, particularly using CO_2_ under supercritical conditions, allows extracts rich in fat-soluble compounds such as carotenoids and tocopherols to be obtained, minimising exposure to oxygen and high temperatures [246,247,248]. Ultrasound-assisted or microwave-assisted extraction has been shown to increase polyphenol extraction yields by 30–50% and 40–60%, respectively, compared to conventional methods, while reducing process times and solvent use [249,250,251].

Freeze-drying is one of the most effective technologies for the long-term preservation of antioxidant-rich products [252]. The removal of water by sublimation at low temperatures and under vacuum preserves the molecular structure of heat-sensitive compounds, maintaining up to 90–95% of the original antioxidant activity [253]. However, high energy costs limit its large-scale application. Emerging technologies such as cold plasma treatment at atmospheric pressure and microencapsulation have shown significant potential in improving the bioaccessibility and antioxidant properties of bioactive compounds [254,255]. Spray drying encapsulation has been found to be one of the most effective technologies for minimising oxidative deterioration, although the high temperatures of the traditional process can completely degrade omega-3s if not properly controlled [256]. Studies have shown that electrospray, operating at lower temperatures than conventional spray drying, better preserves omega-3s and achieves higher encapsulation rates when whey proteins or soy protein isolates are used as wall materials [257].

### 8.2. Interactions Between Food Matrix and Antioxidant Molecules

The food matrix plays a fundamental modulating role in the stability, bioaccessibility and bioavailability of antioxidant compounds. The physicochemical interactions between the components of the matrix and bioactive molecules significantly influence their fate during gastrointestinal digestion [258,259]. Ethyl esters, commonly used in supplements, require the action of pancreatic carboxyl esterase to be hydrolysed, an enzyme whose activity is enhanced by the presence of dietary fats [260]. The bioavailability of ethyl esters is significantly reduced when taken on an empty stomach, making it advisable to take them with food. On the contrary, omega-3 in the form of phospholipids show high bioavailability even in the absence of fat-rich meals, thanks to different blood transport routes and hepatic metabolism [261].

Dietary lipids are a critical factor in the bioavailability of fat-soluble antioxidant. The presence of fats in the food matrix stimulates the secretion of bile salts and pancreatic lipase, promoting the formation of mixed micelles that incorporate carotenoids, tocopherols and other lipophilic compounds [262]. Bioavailability studies have shown that the absorption of lycopene from tomatoes increases 2–5 times when consumed together with lipid sources, with a dose-dependent effect [263]. The bio isomerisation of lycopene from trans to cis during heat treatment can also increase its bioavailability, as cis isomers are more easily solubilised in acidic bile micelles [264].

Dietary proteins can form complexes with polyphenols through non-covalent interactions, mainly hydrogen bonds and hydrophobic interactions. These polyphenol-protein complexes can modify the bioaccessibility of phenolic compounds in two ways: on the one hand, they can protect polyphenols from degradation during gastrointestinal transit, and on the other, they can temporarily reduce their release from the matrix [265,266]. Casein and whey proteins have shown particular affinity for tea catechins, modulating their release profile during simulated in vitro digestion [267].

Complex carbohydrates and dietary fibre significantly influence the release kinetics of bioactive compounds. Pectin and other polysaccharides can physically trap antioxidant molecules, slowing their release into the gastric and intestinal environment [268]. Although this phenomenon reduces the rate of absorption, it can protect sensitive compounds from the acidic gastric environment and prolong their stay in the intestinal tract, promoting absorption in the distal portions of the small intestine [269]. Recent studies have shown that co-administration of polyphenols with inulin and galacto-oligosaccharides can positively modulate the composition of the intestinal microbiota and the production of bioactive microbial metabolites [270,271]

Antioxidant-antioxidant interactions represent an additional level of complexity. Synergistic effects have been documented between vitamin C and vitamin E, where ascorbic acid is able to regenerate the reduced form of tocopherol after it has neutralised free radicals. Similarly, flavonoids can exert protective effects against β-carotene, preventing its oxidation under pro-oxidant conditions [272,273].

### 8.3. Nutraceutical Formulations to Improve Bioavailability

The inherent limitations of natural antioxidant compounds, such as low water solubility, chemical instability and poor intestinal absorption, have stimulated the development of delivery systems designed to improve their bioavailability and biological efficacy.

Microencapsulation by freeze-drying is a particularly effective alternative for preserving the oxidative stability of omega-3s. Studies on microcapsules containing a blend of chia oil (50%) and fish oil (50%) have shown that optimising operating conditions (air inlet temperature, wall material concentration, pump speed) can reduce losses of alpha-linolenic, eicosapentaenoic and docosahexaenoic acid to less than 5%, while maintaining very low peroxide values [274].

Liposomes are vesicular systems consisting of phospholipid bilayers that enclose an internal aqueous compartment. This structure allows the encapsulation of both hydrophilic molecules in the aqueous core and lipophilic compounds in the phospholipid bilayer [275]. Quercetin liposomes have been shown to increase oral bioavailability by up to 3–4 times compared to the free form, protecting it from degradation in the acidic gastric environment and facilitating its absorption through the intestinal epithelium [276]. Huang et al. (2019) reported encapsulation efficiencies of 93% for resveratrol and 100% for curcumin in multilamellar liposomes, with significantly improved stability during simulated digestion [277].

Lipid nanocarriers, solid lipid nanoparticles (SLNs) and nanostructured lipid carriers (NLCs), offer advantages in terms of physical stability and loading. SLNs consist of lipids that are solid at body temperature, while NLCs combine solid and liquid lipids to create a less ordered matrix that allows for greater encapsulation of active compounds. Resveratrol incorporated into NLCs has shown a 5–8-fold increase in bioavailability compared to suspension administration, with a significant reduction in pre-systemic metabolisation [278]. SLNs associated with phenolic compounds have demonstrated high technological stability during drying processes and prolonged storage [279].

Oil-in-water nanoemulsions are kinetically stable colloidal systems with lipid droplet sizes in the range of 20–200 nm. The reduced size of the droplets significantly increases the surface area available for pancreatic lipase action, accelerating intestinal lipolysis and the formation of mixed micelles [280]. Curcumin delivered in nanoemulsions has shown an improvement in bioaccessibility of up to 70–80% in in vitro digestion models, compared to 5–10% for powdered curcumin [281]. Coconut oil-based nanoemulsions have demonstrated effective brain targeting for intranasal administration of resveratrol in animal models [282]. Nanoemulsions also significantly improve the oxidative stability of omega-3s by creating microenvironments with reduced exposure to oxygen [283]. The use of natural emulsifiers such as *Quillaja saponaria* saponins can produce nanoemulsions with oxidative stability equivalent to or superior to synthetic emulsifiers, responding to growing consumer demand for products with cleaner labels [284]. Furthermore, the incorporation of fat-soluble antioxidants (carotenoids, tocopherols) in the oil phase and water-soluble antioxidants (phenolic compounds) in the aqueous phase provides synergistic protection against oxidation [285].

Polymeric systems, such as chitosan or poly-lactic-co-glycolic acid (PLGA) nanoparticles, offer versatility in modulating controlled release. PLGA is particularly interesting due to its biodegradability and biocompatibility [286]. Encapsulating green tea catechins in PLGA nanoparticles has been shown to protect these compounds from intestinal alkaline degradation, prolonging their systemic retention and improving their tissue accumulation [287]. PLGA nanoparticles associated with resveratrol have been shown to be effective in crossing the blood–brain barrier and providing neuroprotection in models of neurodegenerative diseases [288]. Polymeric nanoparticles based on plant proteins such as pea protein isolate have been shown to significantly enhance the ABTS and DPPH radical scavenging activity of curcumin, quercetin, and resveratrol [289].

Cyclodextrins are cyclic oligosaccharides with an internal hydrophobic cavity and a hydrophilic external surface, capable of forming inclusion complexes with lipophilic molecules. Complexation with β-cyclodextrin or its water-soluble derivatives can dramatically increase the aqueous solubility of poorly soluble compounds. Curcumin complexed with hydroxypropyl-β-cyclodextrin showed an increase in solubility of over 1000 times, resulting in improved oral bioavailability [290].

The use of non-lamellar liquid crystals (cubic, hexagonal, sponge phase) has shown potential for use as delivery systems in the food, pharmaceutical and cosmetic industries [291]. Their combination with microencapsulation using antioxidant-rich plants improved the oxidative stability of omega-3s in powder form [292].

**Figure 5:**
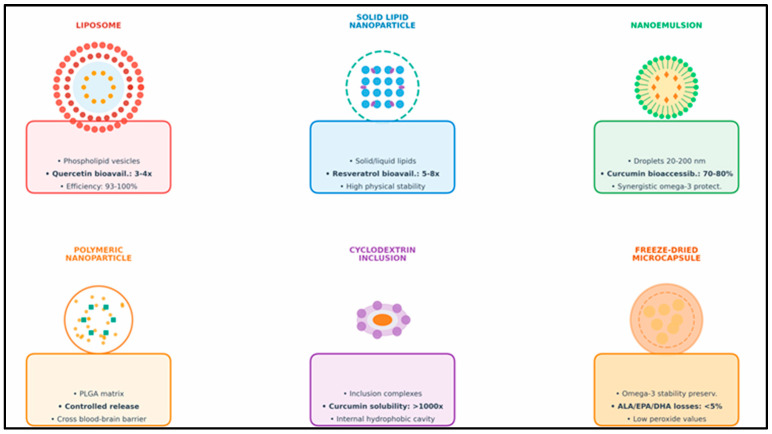
Schematic representation of the main delivery systems used in food systems.

Figure 5 summarises the main characteristics of the delivery systems used for bioactive molecules in the food sector.

## 9. Conclusions

Current evidence identifies aging as a dynamic and plastic biological process rather than an inexorable decline. Within this framework, nutritional and nutraceutical strategies serve as pivotal modulators of the aging trajectory. Caloric restriction remains the leading method for promoting longevity. By suppressing the mTOR pathway, it strengthens the body’s antioxidant defenses and reduces age-related chronic inflammation [161]. Moreover, chrononutrition strategies, such as Intermittent Fasting and Time-Restricted Eating, promote autophagy and robust cellular repair mechanisms [166]. Qualitative dietary patterns, such as the Mediterranean, Plant-Based, and DASH diets, mitigate systemic inflammation and oxidative stress [172,173]. Their efficacy relies on a high density of vitamins and minerals, which act as essential enzymatic cofactors to maintain genomic stability and preserve intrinsic capacity [197].

Beyond broad dietary patterns, the targeted integration of specific bioactive compounds and nutraceuticals represents a precision strategy for addressing the discrete molecular hallmarks of aging. Polyphenols enhance mitochondrial quality and stabilize the epigenome by modulating key metabolic axes, including SIRT1/AMPK, Nrf2/PGC-1α, and NF-κB/NLRP3 [216].

Curcumin exhibits a broad spectrum of geroprotective effects, particularly by mitigating the pro-inflammatory environment [221]. Meanwhile, carotenoids specifically bolster mitochondrial efficiency and antioxidant resilience. Furthermore, Omega-3 fatty acids, notably EPA and DHA, act as potent modulators of systemic inflammation, membrane fluidity, and gene expression [223]. At the interface of the host-environment interaction, probiotics and postbiotics fortify the intestinal barrier and regulate the gut–brain axis, effectively reducing permeability and the chronic inflammatory burden [229].

The synergy between mitotropic compounds, AMPK/Nrf2 activators, and senolytics targets the crosstalk between mitochondrial decay and inflammaging. This approach preserves epigenetic stability and eubiosis, protecting cardiovascular, metabolic and cognitive health.

The clinical efficacy of these nutritional interventions is fundamentally contingent upon the successful delivery of bioactive molecules to their biological targets. This objective is achieved through the targeted use of advanced food technologies. These technologies enhance bioavailability and ensure precise release kinetics, allowing compounds to bypass the gastric barrier and maximize cellular resilience.

Current research aims to standardize experimental evidence into clinical protocols suitable for diverse populations [293].

Moving toward early-stage detection, advanced biomarkers can identify silent metabolic shifts decades before they manifest [294]. This transforms nutrition from a reactive treatment for frailty into a strategic intervention to maintain intrinsic capacity [295].

At the same time, the field is moving beyond narrow monogenic approaches toward integrated strategies that address the multiple molecular hallmarks of aging simultaneously. The future of longevity science lies at the intersection of nutritional biochemistry, systems biology, and precision medicine. This synergy is essential to decode senescence and implement tailored interventions that prevent chronic disease and ensure lasting functional health.

## Figures and Tables

**Table 1:** Relative contribution of major intracellular sources of ROS during aging.

ROS Source	Subcellular Localization	Conditions	Relevance in Aging	References
**Mitochondrial electron transport chain (Complex I–III)**	Mitochondria	Basal metabolism, aging, mitochondrial dysfunction	Major driver of mtDNA damage; senescence induction	[64,65,66,67]
**NADPH oxidases (NOX1/2/4)**	Plasma membrane, cytosol, ER	Inflammation, inflammaging, senescence	Amplification of inflammation; SASP	[36,67,68,69,70]
**ER oxidoreductases**	Endoplasmic reticulum	ER stress, protein misfolding,	Proteostasis failure	[76,77]
**Mitochondrial enzymes**	Mitochondria	Krebs’ cycle fuelling, lipid metabolism	Membrane damage	[57,58]
**Pro-inflammatory cytokines**	Cytosol mitochondria	Stress response	Local ROS signaling; amplification of inflammation; SASP	[64,65,66,67]

**Table 2:** Major antioxidant systems and therapeutic targets regulating ROS during aging.

Antioxidant System Pathway	Primary ROS Controlled	Age-Associated Alteration	Therapeutic Relevance	References
**Superoxide dismutases (SOD1, SOD2, SOD3)**	O_2_•^−^	Reduced activity with aging	First-line ROS detoxification, preserves mitochondrial integrity	[139,140,141,142,143,144,145,146]
**Catalase**	H_2_O_2_	Compensatory increase or dysfunction	Protection from H_2_O_2_ accumulation	[111]
**Glutathione system (GSH, GPx, GR)**	H_2_O_2_, lipid peroxides	Decline in GSH availability with aging	Central redox buffer, modulated by nutrition	[112,113,114]
**Thioredoxin system (TRX/TrxR)**	Protein thiols	Reduced efficiency in aged tissues	Regulation of redox-sensitive signaling	[112,113]
**NRF2–KEAP1 pathway**	Global oxidative stress	Impaired activation in aging	Master regulator of antioxidant gene expression	[115,116,117]
**AMPK–PGC-1α axis**	Indirect (mtROS reduction)	Reduced activation with aging	Enhances mitochondrial biogenesis and antioxidant defenses	[128,129,130,131,132,133]
**Sirtuins (SIRT1, SIRT6)**	Indirect (NAD^+^-dependent)	Age-related decline in activity	Genome stability, redox homeostasis	[139,140,141,142,143,144,145,146]

## Data Availability

Data are available from the authors upon reasonable request.

## Abbreviations

The following abbreviations are used in this manuscript:

AA	arachidonic acid (20:4n-6)
AMPK	AMP-activated protein kinase
AREs	Antioxidant Response Elements
BCKDH	branched-chain ketoacid dehydrogenase
BDNF	brain-derived neurotrophic factor
CDKIs	cyclin-dependent kinase inhibitors
COX	cyclooxygenase
CR	Calorie restriction
CRMs	calorie restriction mimetics
CRP	C-reactive protein
DAMPs	damage-associated molecular patterns
DASH	hypertension diet
DDR	DNA damage response
DHA	docosahexaenoic acid (22:6n-3)
DNMTs	DNA methyltransferases
DPA	docosapentaenoic acid
eNOS	endothelial nitric oxide synthase
EPA	eicosapentaenoic acid (20:5n-3)
EFOX	electrophilic oxo-derivatives
ER	endoplasmic reticulum
FOXO	Forkhead Box O
GPx	Glutathione peroxidases
GSH	glutathione
GSSG	glutathione disulfide
GST	glutathione S-transferase
H2O2	hydrogen peroxide
HATs	histone acetyltransferases
HDACs	histone deacetylases
HMTs	histone methyltransferases
HO-1	heme oxygenase-1
HSCs	hematopoietic stem cells
IF	Intermittent fasting
IL-6	interleukin-6
KEAP1	Kelch-like ECH-associated protein 1
KGDH	α-ketoglutarate dehydrogenase
LOX	lipoxygenase
LPS	lipopolysaccharide
LTB4	leukotriene B_4_
MaR1	maresins
MCI	mild cognitive impairment
MD	Mediterranean diet
MDA	malondialdehyde
miRNA	microRNA
n-3 LC-PUFAs	Omega-3 long-chain polyunsaturated fatty acids
NADPH	nicotinamide adenine dinucleotide phosphate (reduced form)
NLCs	nanostructured lipid carriers
NO	nitric oxide
NOX	NADPH oxidase
NPD1	neuroprotectins
NQO1	NAD(P)H quinone oxidoreductase-1
NRF2	Nuclear factor erythroid 2-related factor 2
O2	molecular oxygen
O2.-	superoxide
PBD	plant-based diet
PD1	protectins
PDH	pyruvate dehydrogenase
PGC-1α	peroxisome proliferator-activated receptor gamma coactivator 1-alpha
PGE2	prostaglandin E_2_
PLGA	poly-lactic-co-glycolic acid
PRC2	Polycomb Repressive Complex 2
ROS/RNS	reactive oxygen/nitrogen species
RvD1	resolvins
RvE1	resolvins
SA-β-Gal	senescence-associated β-galactosidase
SAH	S-adenosyl-L-homocysteine
SAM	S-adenosyl-L-methionine
SASP	senescence-associated secretory phenotype
SCFAs	short-chain fatty acids
SLNs	solid lipid nanoparticles
SOD2	mitochondrial superoxide dismutases
TAC	antioxidant capacity
TNF-α	tumor necrosis factor-alpha
TNFR2	tumor necrosis factor receptor-2
TRE	time-restricted eating
TRX	thioredoxin
TrxR	thioredoxin reductase
UCPs	uncoupling proteins
XO	xanthine oxidase
5hmC	5-hydroxymethylcytosine
5mC	5-methylcytosine
